# Transcription Profiling of Glycosyltransferases in Vitis vinifera Cultivars Following Smoke Exposure

**DOI:** 10.1021/acs.jafc.5c06076

**Published:** 2025-08-19

**Authors:** Lieke van der Hulst, Lina Herliana, Ali Saleh Hassan, Julian G. Schwerdt, Neil Shirley, Christopher M. Ford, Rachel A. Burton, Kerry L. Wilkinson

**Affiliations:** † School of Agriculture, Food and Wine, Waite Research Institute, 1066 The University of Adelaide, PMB 1, Glen Osmond, Adelaide, South Australia 5064, Australia; ‡ The Australian Research Council Training Centre for Innovative Wine Production, Glen Osmond, Adelaide, South Australia 5064, Australia; § Research Center for Genetic Engineering, National Research and Innovation Agency (BRIN), Bogor, West Java 16911, Indonesia; ∥ The Australian Research Council Centre of Excellence in Plant Cell Walls, PMB 1, Glen Osmond, Adelaide, South Australia 5064, Australia

**Keywords:** smoke taint, grapes, RNA-seq, GO pathway enrichment, glycosyltransferases, GT1, GT8, volatile phenols, *Vitis vinifera*, HSP20s, xenobiotics

## Abstract

Smoke taint is a fault that can occur in wines made from smoke-affected grapes. Smoke taint research has largely focused on chemical and sensory analysis and mitigation strategies, with limited attention to underlying molecular responses. We performed RNA sequencing on berries from Vitis vinifera cultivars Shiraz and Chardonnay after controlled smoke exposure. Significant transcript upregulation was observed in smoke-affected grapes, especially for heat shock proteins (HSP20s) and glycosyltransferases (GTs). Chardonnay showed upregulation of 15 GTs from GT1, GT8, GT32, GT47, and GT90, while Shiraz had only five upregulated GTs, primarily GT1 and GT8, suggesting cultivar-specific responses. Gene Ontology enrichment indicated activation of phenylpropanoid and xenobiotic response pathways. While prior studies focused on GT1, our results highlight potential roles for other GT families, particularly GT8. These findings expand our understanding of grapevine stress responses to smoke and identify new molecular targets to inform future strategies for managing smoke taint in grape production.

## Graphical abstract

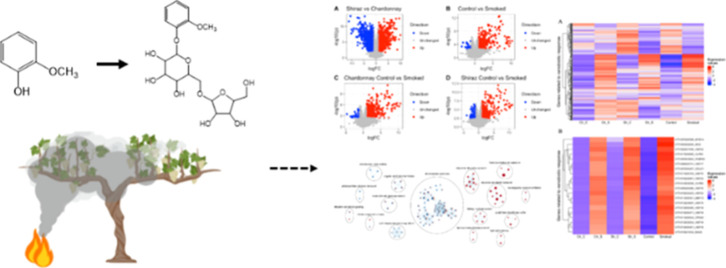

## Introduction

An increase in the frequency of hotter and drier weather patterns has led to a higher incidence of wildfires around the world.^1^ Consequently, countries including Australia, Canada, Chile, South Africa, and the USA have seen bushfires occur in proximity to wine regions in recent years.^2–4^ These fires not only impose economic stress on grapegrowers and winemakers in terms of vineyard management, grape production and reduced cellar-door traffic, but the development of ‘smoke taint’ in wines made from smoke-affected fruit has become a pressing issue.^2,3^ The undesirable smoky and ashy characters evident in some smoke-tainted wines can render the end product unsaleable.^3–5^ The chemical and sensory profiles of wines made following the exposure of grapes or grapevines to smoke have been well studied,^3–9^ but the molecular and biochemical pathways underpinning the development of smoke taint in grapes have not yet been sufficiently investigated.^2,10,11^

The chemical profile of smoke taint has been attributed to the uptake of smoke-derived volatile phenols by grapes, which can subsequently impart various smoky, burnt rubber, medicinal, and ashy aromas and flavors to wine.^3–5^ However, volatile phenols have been shown to accumulate in smoke-affected berries in glycoconjugate forms.^4–9^ While some of these precursors are hydrolyzed during winemaking, causing the release of volatile phenols into wine, and intensification of smoke-related sensory attributes, a substantial proportion of the glycoconjugate pool remains intact in finished wines.^5,8–12^ The glycosylated precursors have been found to be relatively stable in wine over time,^13^ but are also thought to contribute to the sensory perception of smoke taint, since they can be hydrolyzed by enzymes present in human saliva, releasing their volatile aglycone components.^4^

The formation of glycoconjugates of specialized metabolites during plant development and of xenobiotic compounds absorbed from the environment is a common metabolic strategy in plants. Addition of one or more sugars facilitates the solubility and transport of the glycoconjugate and provides a convenient form of storage for small, lipophilic molecules in the cytosol of plant cells.^14^ In their aglycone forms these specialized metabolites are often involved in stress responses, interactions with pollinators and/or general plant defense.^15^

In berries of the cultivated grapevine Vitis vinifera, grape-derived volatile compounds, e.g., norisoprenoids and monoterpenoids, are known to accumulate during berry development and/or ripening as glycosides, contributing to the nonaromatic pool of potential aroma and flavor compounds.^16,17^ More than 200 aglycone glycoside acceptors have been identified in grapevine tissues, with glucosides (where a single sugar is attached at one position on the aglycone acceptor molecule) and diglycosides (two sugar molecules are attached at separate positions on the aglycone molecule) being the most commonly described glycoconjugate forms present in all grape varieties.^18^ Higher order glycosides that feature further substitution of the aglycone-linked sugar (normally glucose) with additional monosaccharides have not been identified for endogenous compounds in grapes, but have been detected in apples and tomatoes.^19,20^ In these crops, higher-order glycoconjugation leads to the accumulation of nonaromatic compounds in ripening fruit.^20^ Higher-order glycosides, including glucosyl-glucosides and rutinosides (rhamnosyl-glucosides), alongside glucosides, have been among the glycoconjugates of volatile phenols detected in grapes following smoke exposure, and in wine made from smoke-affected grapes.^4–9^ More recently, several volatile phenol trisaccharides have also been identified.^12^

Glycosyltransferases (GTs), the enzymes associated with the formation of glycosidic linkages, including the biosynthesis of glycosides, have been the subject of detailed reviews in which the links between structural biology, reaction chemistry and substrate specificity are described.^21^ The CAZy database, http://www.cazy.org, lists over 130 families of GTs. Enzymes associated with the glycosylation of small molecules in plants are commonly categorized in family 1. The sugar, glucose, is derived from UDP-Glucose and hence these enzymes are commonly abbreviated to UGTs. Härtl et al. (2017)^22^ identified three highly expressed UGTs from grapevine berries and leaves as potential candidates implicated in the glucosylation of smoke-derived volatile phenols. The three candidate genes (*UGT88F12*, *UGT72B27* and *UGT92G6*) were expressed in bacteria and enzyme activities tested with a range of endogenous and xenobiotic substrates. Two of the three candidate enzymes, UGT72B27 and UGT92G6, were active in the glucosylation of a number of phenolic aglycones found in smoke, including guaiacol, 4-methylguaiacol, *m*-cresol, syringol, and 4-methylsyringol.^22^ Detailed analysis of the properties of these enzymes showed UGT72B27 had the highest specificity constant for guaiacol (*K*_cat_ */K*_m_ 114.41 M^–1^ s^–1^), and that its endogenous substrate was most likely the stilbene *trans*-resveratrol (*K*_cat_ */K*_m_ 16.97 M^–1^ s^–1^).^22^ UGT92G6 did not form glucosides of *trans*-resveratrol, but was subsequently shown to have a range of activities including the ability to form geraniol diglucoside from geraniol glucoside in vitro (*K*_cat_ */K*_m_ 30 M^–1^ s^–1^).^22^ Geraniol itself was not a substrate for this enzyme, and the formation of geraniol diglucoside was not seen when the gene was used to transform Nicotiana benthamiana plants.^23^ Further work identified at least 4 members of the *UGT85A* subfamily in grapevine with glycosylating activity against volatile phenol substrates; none of the substrates tested, however is believed to be among those found in smoke.^24^

Recently, two papers^10,11^ have examined glycosyltransferase (GT) responses to smoke in grapevines from RNA-seq data. A molecular analysis of V. vinifera cv. Merlot exposed to barbecue smoke pellets indicated that UGTs were among a large number of genes whose expression was upregulated in pre- and postveraison grapes.^10^ Additionally, Paineau et al. (2025)^11^ studied the dwarf grapevine cultivar Pixie and identified 12 GT1 genes significantly upregulated by smoke, naming them V. vinifera smoke-inducible UGTs (*VviSIUGTs*). However, it remains unclear whether other cultivars, such as Chardonnay and Shiraz, exhibit similar GT expression patterns when exposed to smoke, as observed in previous studies. The present study examined the effects of smoke treatment on berries taken from potted grapevines. Molecular analyses were conducted to identify those genes showing differential transcription following exposure to smoke, and the levels of transcription of a number of GTs were compared using RNA-sequencing analysis.

## Materials and Methods

### Potted Grapevines

Two V. vinifera cultivars, Chardonnay and Shiraz, were grown as potted vines in a controlled environment from cuttings taken from a South Australian vineyard (located at the University of Adelaide’s Waite Campus in Urrbrae, 34°58′S, 138°38′E), from May to December 2015, as previously described.^25^ A diurnal rhythm was mimicked by creating 16 h “days” with artificial daylight (intensity 400 μmol photons/m^2^/s) at 27 °C and 8 h “nights” without lighting at 22 °C. The humidity in the growth room was maintained at 35% using a dehumidifier (SECCO ULTRA, Applied Climate Control Pty Ltd., Sydney, NSW, Australia). Prior to smoke exposure, potted vines were moved to an area outside the growth chamber to ensure control vines were not affected. At the time of smoke exposure, the growth room temperature was 27 °C, while outside it was 28 °C. Grapevines were exposed to smoke for 1 h, approximately 2 weeks postveraison, using a purpose-built smoke tent (6.0 × 2.5 × 2.0 m), and methodology described previously.^5^ Smoke treatments were conducted (in triplicate), with each experimental replicate comprising three potted vines. During smoke exposure, air temperature was monitored by placing a temperature tracker (010-11092-30; Garmin, Olathe, KS, USA) among the potted vines, but ≤2 °C increases in temperature were observed relative to ambient conditions (data not shown). Following treatment, smoke-exposed vines were returned to the controlled environment of the growth chamber. To capture the early transcriptional responses to smoke, 1 h after smoke exposure, 10–15 berries were randomly sampled from each control and smoke-exposed treatment replicate (i.e., 3–5 berries from each potted vine) and flash-frozen in liquid nitrogen prior to storage at −80 °C.

### Total RNA Extraction for RNA-seq

Whole berry samples (skin, pulp and seeds) from each replicate were ground in liquid nitrogen and an aliquot (100 mg) of frozen powder subsampled for RNA extraction using a Spectrum plant total RNA kit (Sigma-Aldrich, North Ryde, NSW, Australia), following the manufacturer’s protocol for samples with high water and sugar content. Samples were eluted from the binding column in one step, with multiple eluents from each replicate pooled following quality control on a 1% agarose gel (clear bands for both 18S and 28S RNA), in addition to RNA concentration being measured using a Qubit (Invitrogen Qubit 1.0 Fluorometer Q32857, Turner Biosystems, Sunnyvale, CA, USA).

### Illumina RNA Sequencing

RNA extracted from grape samples was processed at the Australian Genome Research Facility (AGRF, Melbourne, Vic., Australia) for RNA sequencing. Quality control was performed prior to sequencing to determine RNA Integrity Numbers (RIN) and concentration for each sample; samples all presented an RIN of 9 or higher. Stranded RNA libraries were constructed for each biological replicate, with 100 bp single-end reads being sequenced on an Illumina HiSeq 2500 using a HiSeq SR Cluster Kit v4 and SBS Kit V4 (Table S1). An average of 18,138,277 reads were obtained for each sample.

### Functional Annotation

The V. vinifera reference genome PN40024.V4 (May 2021, database version 108.4) was downloaded from https://plants.ensembl.org/Vitis_vinifera/Info/Annotation/#assembly. This genome was predicted to contain 35,134 protein-coding genes and 41,097 transcripts. All protein sequences were used to generate lists of putative functions or protein products (File S1) using BLAST v2.13.0 and MAKER v2.31.11 (“maker_functional_gff”) against the UniProtKb database (https://www.uniprot.org/taxonomy/33090). Other functional annotations, including for CAZy (Carbohydrate-active enzymes) families, PFAM (protein families) and Gene Ontology (GO) terms (Files S2 & S3) were assigned by submitting V. vinifera protein sequences to the eggNOG-mapper Web site (http://eggnog-mapper.embl.de).^26^ The number of genes belonging to different types of glycosyltransferases (GTs) was calculated based on the CAZy annotations and summarized in File S4. To identify orthologous genes, protein sequences from V. vinifera PN40024.V4, Arabidopsis thaliana TAIR10 v20101214 (https://www.arabidopsis.org), and Pyrus × bretschneideri genome v1.1 (https://www.rosaceae.org) were analyzed using OrthoFinder v2.5.5 (File S5).

### RNA-seq Analysis

The RNA-seq data were processed following the pipeline and tools described and referenced in Herliana et al. (2023),^27^ i.e., by quality checking, trimming, cleaning, and aligning reads to the reference genome (Table S2). Quality checking was performed using FastQC v0.11.9 and MultiQC v1.9 with default parameters. Trimmomatic v0.39 removed adapters and PCR primer fragments. BBDuk, BBmap v38.87 was used to remove reads originating from ribosomal (rRNA) genes. The following databases were used as references for rRNA removal, including SILVA (https://www.arb-silva.de) and 5S rRNA (http://combio.pl/rrna) (Table S2). Approximately 1.8–3.6% of the reads were removed, from 14.2–21.8 million reads to 13.8–21.2 million reads (Table S3). Clean reads were aligned to the reference genome with STAR v2.7.10b using a 2-pass procedure. About 95.25% of reads were uniquely mapped to the reference (Table S3). After counting reads with gene-level summarization using featureCounts (subreads v2.01) with the -s 1 parameter to reflect stranded library type, edgeR was used to perform the first step in differential expression analysis. The Trimmed Mean of *M*-values (TMM) normalization was applied to adjust for compositional differences between RNA-seq libraries prior to differential expression analysis. After TMM normalization, Counts Per Million (CPM) were calculated to make expression values more interpretable by scaling the counts relative to the total number of reads, making expression levels comparable across samples. Only genes with CPM >1, expressed in more than four samples for any combination of genotypes (File S6) and treatments, were kept for cluster analysis using the factoextra R package (PCA = Principal Component Analysis). The same parameters were applied for finding differentially expressed genes (DEGs) with limma and for identifying enriched gene sets using Gene Set Enrichment Analysis (GSEA). Using the limma package, the list of DEGs was obtained by applying the Benjamini and Hochberg (BH) correction of the *p*-values to reduce false positives and sorting by *p*-values. Significant DEGs were determined according to the adjusted *p*-values (FDR <0.05) and an absolute value of the log-fold change (log FC) > 1 (Tables 1 and 2, File S7). VennDiagram was used to visualize the number of DEGs.

**Table 1:** 25 Highest Differentially Expressed Genes in Smoke-Affected Versus Control Grapes, Based on Log Fold Change (Log FC) in Shiraz (Sh) and Chardonnay (Ch)

gene identifier	putative protein product	PFAM	Sh	Ch
*Vitvi13g00409*	HSP18.2 18.2 kDa class I heat shock protein	HSP20	11.02	9.60
*Vitvi04g01796*	HSP17.9-D 17.9 kDa class II heat shock protein	HSP20	10.66	8.32
*Vitvi13g02017*	HSP18.2 18.2 kDa class I heat shock protein	HSP20	10.52	8.91
*Vitvi13g02014*	HSP18.2 18.2 kDa class I heat shock protein	HSP20	10.20	9.00
*Vitvi13g00405*	HSP18.2 18.2 kDa class I heat shock protein	HSP20	10.02	8.24
*Vitvi13g02016*	HSP18.2 18.2 kDa class I heat shock protein	HSP20	9.94	8.22
*Vitvi04g01793*	HSP17.9-D 17.9 kDa class II heat shock protein	HSP20	9.32	8.86
*Vitvi04g01797*	HSP17.9-D 17.9 kDa class II heat shock protein	HSP20	9.20	8.44
*Vitvi19g01760*	HSP17.6C 17.6 kDa class I heat shock protein 3	HSP20	9.07	8.12
*Vitvi04g01799*	HSP17.9-D 17.9 kDa class II heat shock protein	HSP20	8.99	9.25
*Vitvi13g00410*	HSP18.2 18.2 kDa class I heat shock protein	HSP20	8.84	7.67
*Vitvi08g00731*	HSP17.3-B 17.3 kDa class I heat shock protein	HSP20	8.57	7.97
*Vitvi04g04041*	HSP17.9-D 17.9 kDa class II heat shock protein	HSP20	8.56	10.53
*Vitvi04g01795*	HSP17.9-D 17.9 kDa class II heat shock protein	HSP20	8.50	9.61
*Vitvi04g01802*	HSP17.9-D 17.9 kDa class II heat shock protein	HSP20	8.27	7.05
*Vitvi04g01794*	HSP17.9-D 17.9 kDa class II heat shock protein	HSP20	7.46	10.17
*Vitvi13g00490*	HSP18.2 18.2 kDa class I heat shock protein	HSP20	7.32	6.82
*Vitvi13g00471*	HSP18.2 18.2 kDa class I heat shock protein	HSP20	7.10	6.08
*Vitvi07g00457*	GOLS1 galactinol synthase 1	Glyco_transf_8	7.05	7.48
*Vitvi01g01512*	HSP21 heat shock protein 21	HSP20	6.82	6.74
*Vitvi18g02138*	OPR11 putative 12-oxophytodienoate reductase 11	Oxidored_FMN	6.72	5.71
*Vitvi04g00135*	HSP17.9-D 17.9 kDa class II heat shock protein	HSP20	6.57	7.16
*Vitvi14g00169*	HSP26-A probable glutathione *S*-transferase	GST_C	6.54	6.83
*Vitvi04g01792*	HSP17.9-D 17.9 kDa class II heat shock protein	HSP20	6.40	6.91
*Vitvi04g04040*	HSP17.9-D 17.9 kDa class II heat shock protein	HSP20	6.34	7.28

**Table 2:** Downregulated Genes in Smoke-Affected versus Control Grapes Based on Log Fold Change, (Log FC) in Shiraz (Sh) and Chardonnay (Ch)

gene identifier	putative protein product	PFAM	Sh	Ch
*Vitvi18g02819*	EXL3 GDSL esterase/lipase EXL3	Lipase_GDSL	–3.89	–2.65
*Vitvi18g01148*	ABCB13 ABC transporter B family member 13	ABC_membrane,ABC_tran	–2.35	–2.18
*Vitvi10g01915*	CSE Caffeoylshikimate esterase	Hydrolase_4	–2.23	–2.27
*Vitvi10g00940*	AAE14 2-succinylbenzoate--CoA ligase	AMP-binding,AMP-binding_C	–2.14	–2.12
*Vitvi13g00786*	PDR2 Pleiotropic drug resistance protein 2	ABC2_membrane,ABC_tran,ABC_trans_N,PDR_assoc	–1.94	–1.40
*Vitvi18g00882*	JGB Protein JINGUBANG	WD40	–1.89	–1.76
*Vitvi01g01460*	CASP-like protein 2A1	DUF588	–1.61	–2.00
*Vitvi18g00881*	Inactive protein kinase	Pkinase,Pkinase_Tyr	–1.28	–1.57
*Vitvi11g00362*	Probable serine incorporator	Serinc	–1.08	–1.14

The list of DEGs as candidate genes in response to smoke treatment (Tables 1–3) was then compared to two previous studies: Hewitt et al. (2024)^10^ (File S8) and Paineau et al. (2025)^11^ (File S9), to identify overlapping genes and assess consistency with previously reported smoke-responsive genes. As Paineau et al. (2025)^11^ used a different reference genome, the only available approach for comparison was to perform a BLASTn search with an *E*-value threshold of 1 × 10^–5^ of the coding sequences of our candidate genes against their database (https://grapegenomics.com).

**Table 3:** Upregulated Glycosyltransferase Genes Based on Log Fold Change (Log FC) in Smoked vs Control Grape Samples of Shiraz (Sh) and Chardonnay (Ch)

gene identifier	putative protein product	PFAM	CAZy	Sh	Ch
*Vitvi07g00457*	galactinol synthase 1	Glyco_transf_8	GT8	7.05	7.48
*Vitvi15g01623*	hydroquinone glucosyltransferase	UDPGT	GT1	4.65	4.98
*Vitvi11g01294*	anthocyanidin 3-*O*-galactosyltransferase	UDPGT	GT1	2.12	1.06
*Vitvi03g00566*	gallate 1-beta-glucosyltransferase	UDPGT	GT1	1.46	1.93
*Vitvi03g00533*	abscisate beta-glucosyltransferase	UDPGT	GT1	1.36	1.70
*Vitvi05g04355*	phloretin 4′-*O*-glucosyltransferase	UDPGT	GT1		6.00
*Vitvi03g04511*	putative UDP-glucose flavonoid 3-*O*-glucosyltransferase 3	UDPGT	GT1		5.23
*Vitvi08g01006*	scopoletin glucosyltransferase	UDPGT	GT1		1.93
*Vitvi08g01349*	uncharacterized protein	Gb3_synth, Gly_transf_sug	GT32		1.91
*Vitvi07g01394*	linamarin synthase 2	UDPGT	GT1		1.91
*Vitvi06g04226*	UDP-glycosyltransferase 87A1	UDPGT	GT1		1.33
*Vitvi13g04129*	7-deoxyloganetic acid glucosyltransferase	UDPGT	GT1		1.27
*Vitvi08g01873*	probable glucuronoxylan glucuronosyltransferase	EGF_2, Exostosin	GT47		1.18
*Vitvi05g02116*	crocetin glucosyltransferase	UDPGT	GT1		1.14
*Vitvi15g01151*	*O*-glucosyltransferase 1	Glyco_transf_90	GT90		1.03

Using the GSEA v4.1.0 desktop application, enrichment analysis was performed on the normalized expression data, not the DEG list. A customized gene sets database (V. vinifera GO term database, File S3) was used, selecting 1000 as the number of permutations without collapsing the data set and using the gene set as permutation type and selecting a paired phenotype. The GSEA results were visualized using the Cytoscape v3.8.0 application with Enrichment Map installed.

### Field-Grown Grapevines

Control and smoke-affected grapes were collected from four V. vinifera cultivars, Cabernet Sauvignon, Chardonnay, Merlot and Sauvignon Blanc, grown at the University of Adelaide’s Waite Campus. Grapevines were planted (in 1998, on their own roots) to a north–south alignment hand-pruned to a two-node spur system and trained to a bilateral cordon, with vertical shoot positioning and drip irrigation. Smoke treatments were applied approximately 1–2 weeks postveraison using the same smoke tent and experimental approach used for smoke exposure of the potted vines. For each cultivar, samples (∼200 berries per replicate, in triplicate) were collected at maturity for determination of phenolic glycosides by HPLC-MS/MS. For gene expression studies, samples were taken at 3 distinct time points: 1 h after smoke exposure (day 0); 24 h after smoke exposure (day 1); and 7 days after smoke exposure (day 7). Samples (∼50 berries) were immediately separated into skin and pulp fractions, to obtain 10 g of each wet tissue per sample. Fractions were then flash-frozen in liquid nitrogen prior to storage at −80 °C.

### Total RNA Extraction and cDNA Synthesis

Total RNA extraction was performed following the same protocol used for RNA sequencing, except for the addition of a DNase digestion on the column, as per the manufacturer’s instructions (Sigma-Aldrich). For cDNA synthesis, two independent reactions were undertaken for each sample. In the first step, 2 to 11 μL of RNA (depending on concentration) was mixed with 1 μL of 50 μM oligo-dT primer, 2 μL of 5 mM dNTP mix and sterile water to a volume of 14.75 μL. The mixture was heated to 65 °C for 5 min, before being immediately cooled on ice. A master mix containing 4 μL 5× First Strand Buffer, 1 μL dithiothreitol, and 0.25 μL SuperScript III was added to each sample for a total volume of 20 μL, before incubating the reaction at 50 °C for 70 min, followed by inactivation at 70 °C for 15 min cDNA was stored at −20 °C for further verification and analysis.^28^

### Real-Time Q-PCR

Real-time Q-PCR was performed on a single bulked replicate (comprising tissue from multiple berries) of each skin and pulp field sample for all four cultivars, due to cost and time constraints. Primers were designed using Primer3 (Table S4) and real-time Q-PCR was performed as previously described.^29^ The following modifications to the method were made. To provide a template for the standard curve, between four and six 20 μL PCR reaction mixtures were combined for purification by HPLC using a HELIX DNA DVB 50–3 3.0 mm monolithic polymer reversed-phase column (Varian). Chromatography was performed using buffer A (100 mM triethylammonium acetate [Applied Biosystems] and 0.1 mM EDTA) and buffer B (100 mM triethylammonium acetate, 0.1 mM EDTA and 75% acetonitrile). The gradient was as follows: time 0 min, 10% buffer B; time 6 min, 21.5% buffer B; time 7 min, 21.5% buffer B; time 8 min, 10% buffer B; time 12 min, 10% buffer B. The flow rate was 0.45 mL/min and the temperature was 50 °C. Three replicates of each of the seven standard concentrations were included with every Q-PCR experiment together with a minimum of three no-template controls. Q-PCR experiments were assembled by the liquid-handling CAS-1200 robot (Corbett Robotics). Three replicate PCRs for each of the cDNAs were included in every run containing: 2 μL of cDNA solution, the diluted standard, or water, 5 μL of IQ SYBR Green PCR reagent (Biorad Laboratories), 1.2 μL of each of the forward and reverse primers at 4 mM, 0.3 μL of 103 SYBR Green in water, and 0.3 μL of water. The total volume of the PCR reactions was 10 μL. Reactions were performed in an RG 6000 Rotor-Gene real-time thermal cycler (Corbett Research): 3 min at 95 °C followed by 45 cycles of 1 s at 95 °C, 1 s at 55 °C, 30 s at 72 °C, and 15 s at the optimal acquisition temperature. Normalization was carried out using the control genes for V. vinifera actin, ubiquitin, tubulin and malate dehydrogenase (primers found in Table S4) and the final concentrations of mRNAs of the genes of interest are expressed as arbitrary units that represent the numbers of copies per microliter of cDNA, normalized against the geometric means of the three control genes that vary the least with respect to each other.^28^

### Compositional Analysis of Grapes

Analysis of the phenolic glycoside precursor pool was performed using HPLC-MS/MS by the Australian Wine Research Institute’s commercial services laboratory (Adelaide, SA, Australia), using a published stable isotope dilution assay.^6^ An aliquot (5 g) of berry homogenate was spiked with *d*_3_-syringol-gentiobioside as an internal standard. The sample was spun down and an aliquot of the supernatant (2 mL) applied to an Extract Clean C18-HF SPE cartridge (500 mg/4 mL, Grace Davison Discovery Sciences, Baulkham Hills, NSW, Australia). The methanol extract was dried and subsequently reconstituted with 0.5 mL of water prior to instrumental analysis using a 4000 Q TRAP hybrid tandem mass spectrometer with a TurboV ion source (Applied Biosystems/MDS SCIEX, Concord, ON, Canada) combined with an Agilent 1200 HPLC system (Agilent Technologies, Forest Hill, Vic., Australia). Aliquots of 10 μL of the extracted samples were injected and a 3 μm Gemini C6-Phenyl 110 Å column was used for chromatographic analysis. The mobile phases consisted of 0.1% acetic acid in water (solvent A) and 0.1% acetic acid in acetonitrile (solvent B), with a linear elution gradient at a flow rate of 300 μL/min. Mass spectra were recorded in negative ion mode and acquisition and processing of the obtained data were performed using Analyst software version 1.5 (Applied Biosystems/MDS SCIEX).

### Data Availability

The data sets generated during this study were deposited in the NCBI SRA (Sequence Read Archive) database under the BioProject ID: PRJNA892716. Transcriptome data are available under accession numbers SRR21996675–SRR21996686.

## Results

### Identification of Differentially Expressed Genes in Berries from Control and Smoke-Exposed Potted Chardonnay and Shiraz Vines

Transcripts from control and smoke-exposed Chardonnay and Shiraz berries were compared by RNA sequencing to define differential gene expression. Samples were obtained from potted vines cultivated in a controlled growth room environment, harvested 1 h after smoke exposure.

Using principal component analysis (PCA), 77.4% of the variation in sample groups was explained (Figure 1A). PC1 (56.1%) differentiated sample groups based on genotype (Chardonnay vs Shiraz), while PC2 (21.3%) separated smoked samples from the control ones. Thus, there are four separate groups, namely, Chardonnay control (Ch_C), Chardonnay smoke (Ch_S), Shiraz control (Sh_C), and Shiraz smoke (Sh_S). The number of differentially expressed genes (DEGs) (Figures 1B and 2) between control and smoked-affected samples in Chardonnay was 686, with 648 upregulated and 38 downregulated. In contrast, 504 DEGs were observed in Shiraz, with 382 upregulated and 122 downregulated. Of note, there were 263 upregulated and only nine down-regulated genes overlapping between these two genotypes. Heat-shock protein (HSP20) genes dominated the list of the most highly upregulated genes (Table 1) following smoke exposure. In contrast, the most highly downregulated genes (Table 2) showed greater functional diversity, including genes involved in lipid metabolism (EXL3 GDSL esterase/lipase), transport processes (ABCB13 ABC transporter, PDR2), cell wall modification (CASP-like protein), signaling pathways (Inactive protein kinase), and secondary metabolite biosynthesis (Caffeoylshikimate esterase, AAE14 2-succinylbenzoate--CoA ligase).

**Figure 1:**
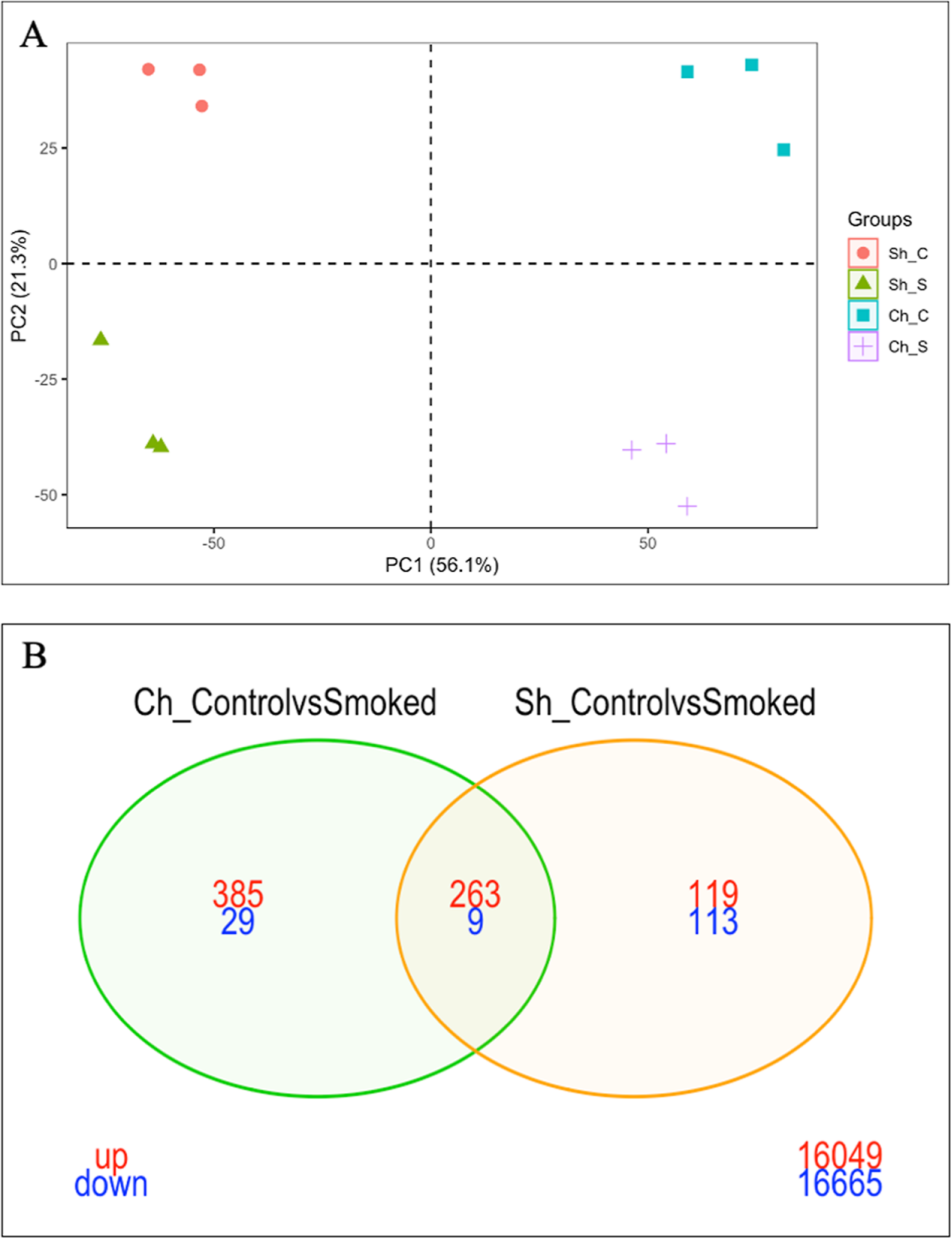
(A) Principal component analysis (PCA) plot of RNA-seq data shows sample clustering according to genotypes and treatments; Sh = shiraz; Ch = chardonnay; C = control; S = smoke-affected samples. (B) Venn diagrams for Differentially Expressed Genes (DEGs) in the two comparison groups. DEGs are grouped into two comparison groups represented by the green circle for treatment comparisons in Chardonnay and the orange circle in Shiraz. The number of DEGs common to the two comparison groups is shown in overlapping portions of the two circles. Upregulated genes are shown in red, while down-regulated genes are shown in blue.

**Figure 2:**
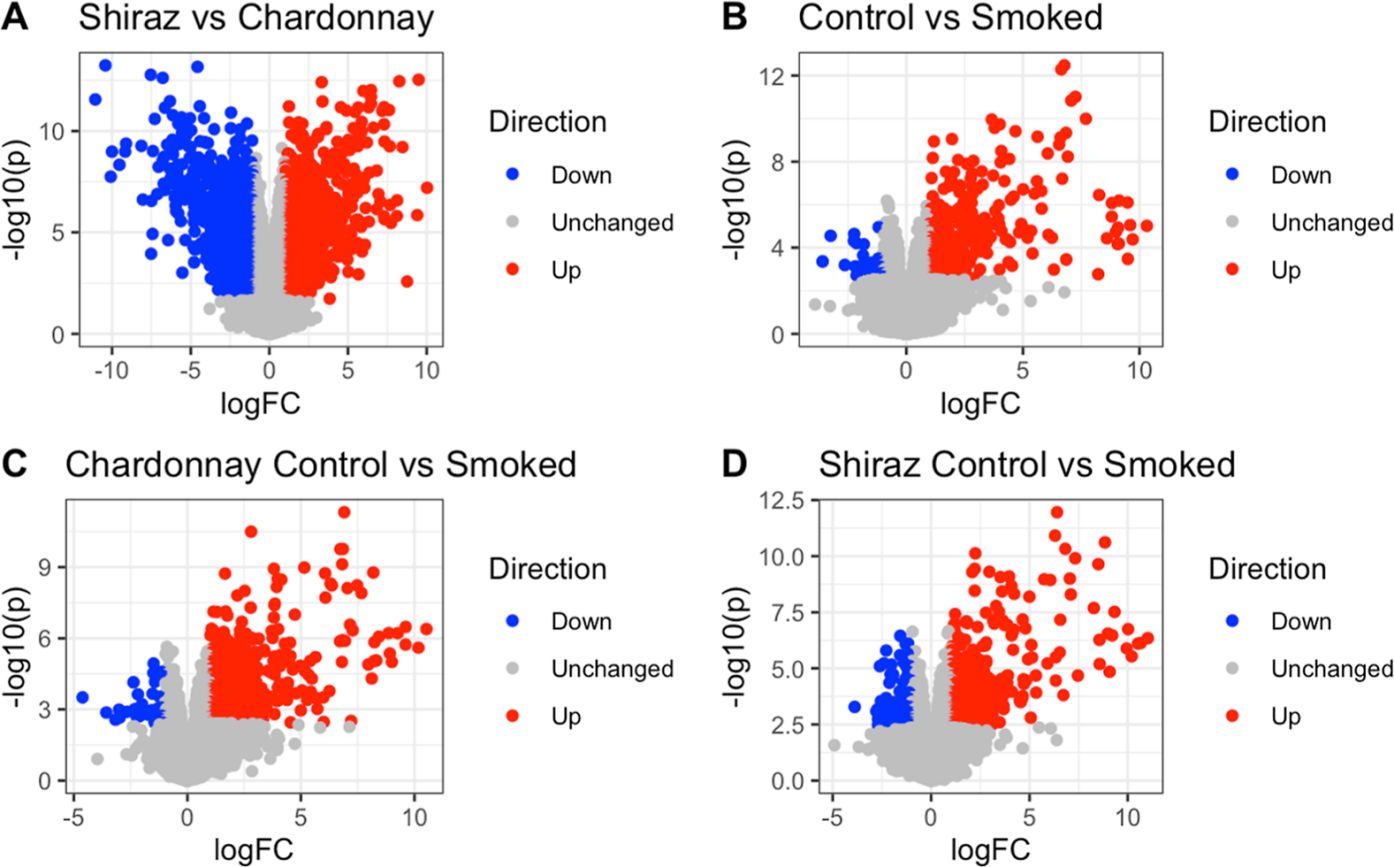
Volcano plots for expressed genes in the four comparison groups. (A) Comparison between two genotypes (Shiraz vs Chardonnay), (B) control vs smoke-affected samples, (C) treatments in Chardonnay, and (D) treatments in Shiraz. Fold changes in gene expression levels (log FC) are shown on the *X* axes, and the significance levels for each gene (−log 10) are shown on the *Y* axes. Each dot represents a single gene. Red dots are representatives of upregulated genes, while down-regulated genes are shown in blue. Gray dots represent genes with unchanged gene expression.

### Identification of Smoke–Responsive Glycosyltransferase Genes

Using eggNOG-mapper v2, 28,847 out of 35,134 (82.11%) genes in the grapevine genome were functionally annotated based on orthology assignments (File S2). From these, about 468 (1.33%) encode glycosyltransferases (GTs), with the GT1 family being the most abundant, comprising 163 genes, followed by the GT8 family with 36 genes (File S4).

In total, 16,816 out of 35,134 genes (47.86%) were expressed in our samples, including 47 GT1, 21 each from GT8 and GT2, 19 GT31, 11 GT4 and 10 genes each from GT48, GT47, and GT14 (File S6). Notably, four GT1 genes and a single GT8 were upregulated in smoked samples, consistently found in both Chardonnay and Shiraz (Table 3, File S7). These genes are *Vitvi03g00533* (AOG Abscisate β-glucosyltransferase), *Vitvi03g00566* (UGT84A13 Gallate 1-β-glucosyltransferase), *Vitvi11g01294* (F3GT1 Anthocyanidin 3-*O*-galactosyltransferase), *Vitvi15g01623* (AS Hydroquinone glucosyltransferase), and *Vitvi07g00457* (GOLS1 Galactinol synthase 1). Among these genes, *Vitvi07g00457* (GOLS1 Galactinol synthase 1, GT8) had the highest fold change of 132.51 (Log FC = 7.05) and 178.53 (Log FC = 7.48) in smoke-treated Chardonnay and Shiraz samples respectively compared to the controls, followed by *Vitvi15g01623* (Hydroquinone glucosyltransferase) showing almost a 32-fold difference (Log FC = 4.65 and 4.98, Table 3) in expression between the smoked and the control samples for both genotypes. Additionally, Chardonnay samples had ten other upregulated genes belonging to the glycosyltransferase family, including seven GT1 genes and one gene from each of the GT32, GT47, and GT90 families. One GT1 gene (*Vitvi05g04355*) encoding a putative UGT75L17 Phloretin 4′-*O*-glucosyltransferase had the highest log FC (6.00) for Chardonnay, but it was not upregulated in smoke-treated Shiraz samples (Table 3, File S7). No expression (zero CPM) was detected in the three replicate control Shiraz samples. However, the data clearly show that this gene was induced by smoke exposure, as evidenced by its expression in all three smoke-treated Shiraz replicates (File S6).

### Selected Glycosyltransferase Transcript Analysis on Field Samples

Transcript levels of three selected glycosyltransferase genes, *Vitvi07g00457* (GOLS1 Galactinol synthase 1, GT8), *Vitvi15g01623* (Hydroquinone glucosyltransferase, GT1), and *Vitvi05g04355* (putative UGT75L17 Phloretin 4′-*O*-glucosyltransferase, GT1) were examined by qPCR in pulp and skin tissues of four grapevine cultivars (Chardonnay, Cabernet Sauvignon, Merlot and Sauvignon Blanc) at 0, 1, and 7 days postsmoke exposure. Fold changes were calculated by comparing transcript levels between smoke-exposed and control samples (Figure 3, Table S5). Among the three genes, *Vitvi07g00457* showed the strongest induction, particularly at day 7, in both tissue types of Cabernet Sauvignon, Chardonnay, and Merlot, suggesting a robust smoke-responsive pattern. In contrast, *Vitvi15g01623* showed early but moderate induction, while *Vitvi05g04355* maintained relatively stable and low expression across all cultivars and time points. Notably, all three genes were either downregulated or unchanged in Sauvignon Blanc, indicating a distinct, cultivar-specific response to smoke exposure. Expression changes were generally more pronounced in skin compared to pulp, suggesting tissue-specific regulation. Collectively, these findings highlight *Vitvi07g00457* as the most responsive gene to smoke exposure, particularly in Cabernet Sauvignon and Merlot, and emphasize the importance of genetic background in modulating transcriptomic responses to environmental stress.

**Figure 3:**
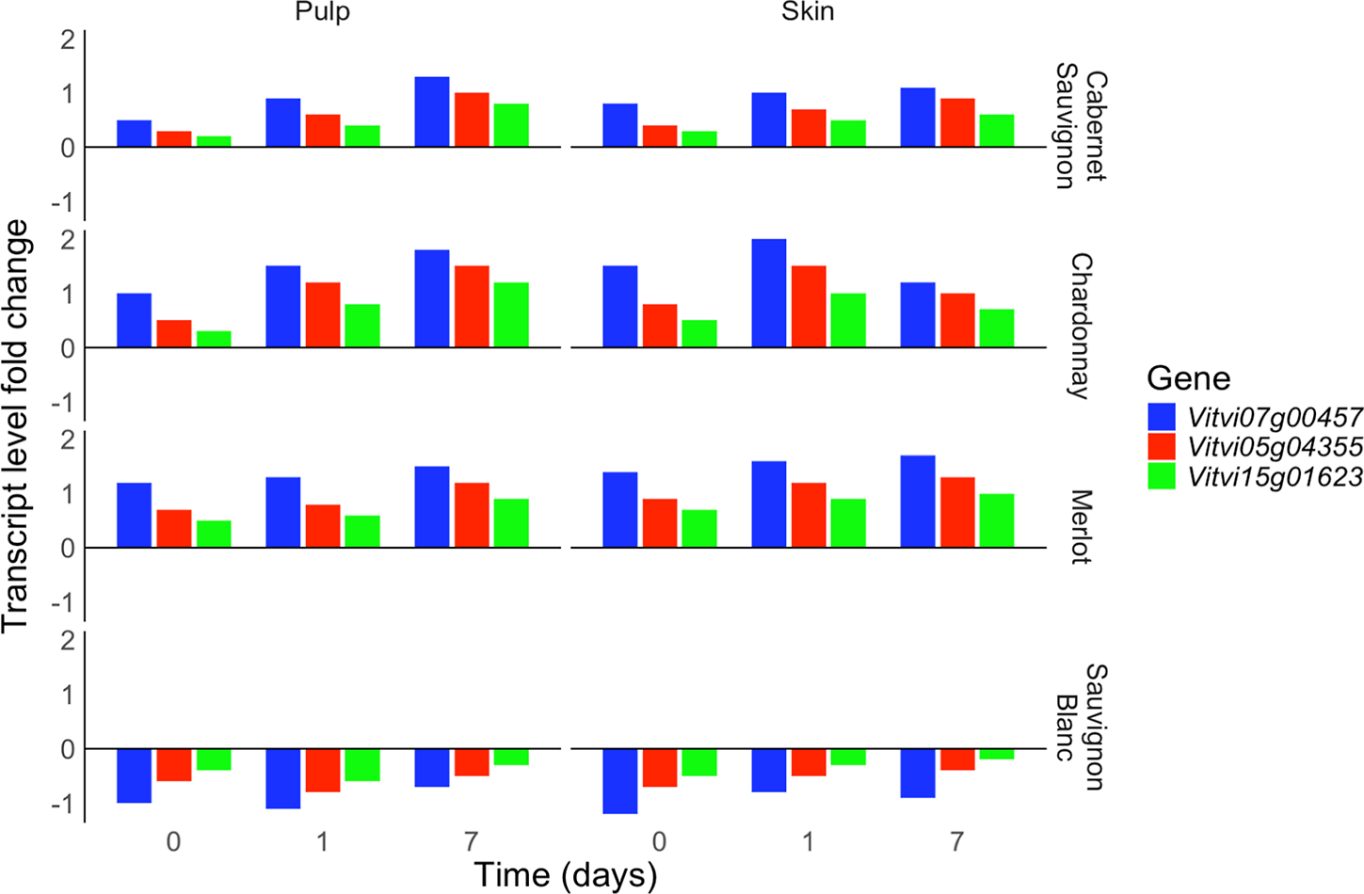
RT-qPCR analysis of Transcript level fold changes between smoke-exposed and control berries of target genes *Vitvi07g00457* (GOLS1 Galactinol synthase 1, GT8) in blue, *Vitvi15g01623* (Hydroquinone glucosyltransferase, GT1) in red, and *Vitvi05g04355* (putative UGT75L17 Phloretin 4′-*O*-glucosyltransferase, GT1) in green over time for Vitis vinifera cv Cabernet Sauvignon, Chardonnay, Merlot and Sauvignon Blanc.

### Identification of Smoke–Responsive Pathways

Gene Set Enrichment Analysis (GSEA) revealed both genotype-dependent and independent upregulated pathways, as defined by enriched GO terms. In this context, genotype-dependent pathways refer to those uniquely enriched in one genotype, while genotype-independent pathways are enriched in both genotypes. A total of 16 significantly enriched pathways were identified, each consisting of more than one GO term within one cluster and showing enrichment in at least one genotype (Table S6). Among these, four clusters were classified as genotype independent, meaning they were common to both genotypes and primarily composed of upregulated genes (Table 4). These included pathways related to xenobiotic transport, heat acclimation temperature, high light intensity, and lignin metabolic phenylpropanoid. The response to xenobiotic transport is the biggest cluster among these four with eight nodes (Figure 4) or GO terms (Table 4). Expression heatmaps of genes related to xenobiotic transport are visualized in Figure 5. The gene expression in each pathway or cluster can be found in Files S10–S13. At least one of the top five upregulated genes in response to the xenobiotic transport cluster is involved in the other three independent pathways. For example, *Vitvi07g00457* (GOLS1 Galactinol synthase 1), *Vitvi08g00731* (HSP17.3-B 17.3 kDa class I heat shock protein), and *Vitvi17g00695* (CLPB1 Chaperone protein ClpB1) were detected in three clusters but not in the lignin metabolic phenylpropanoid. Two other genes, *Vitvi15g01623* (a hydroquinone glucosyltransferase) and *Vitvi13g04087* (HSP15.7 15.7 kDa heat shock protein, peroxisomal), were detected in the lignin metabolic phenylpropanoid cluster and the heat acclimation temperature groups, respectively.

**Table 4:** Clusters of Enriched Gene Ontology (GO) Terms in Smoke-Affected Samples of Both Genotypes

gene ontology (GO) term	definition	number of genes
Response Xenobiotic Transport		
GO:0006855	xenobiotic transmembrane transport	50
GO:0015893	xenobiotic transport	51
GO:0042542	response to hydrogen peroxide	56
GO:0000302	response to reactive oxygen species	126
GO:0009636	response to toxic substance	170
GO:0046677	response to antibiotic	179
GO:0006979	response to oxidative stress	263
GO:0042493	response to xenobiotic stimulus	315
Heat Acclimation Temperature		
GO:0010286	heat acclimation	29
GO:0009408	response to heat	139
GO:0009266	response to temperature stimulus	377
High Light Intensity		
GO:0009644	response to high light intensity	58
GO:0009642	response to light intensity	112
Lignin Metabolic Phenylpropanoid		
GO:0009808	lignin metabolic process	17
GO:0009699	phenylpropanoid biosynthetic process	32

**Figure 4:**
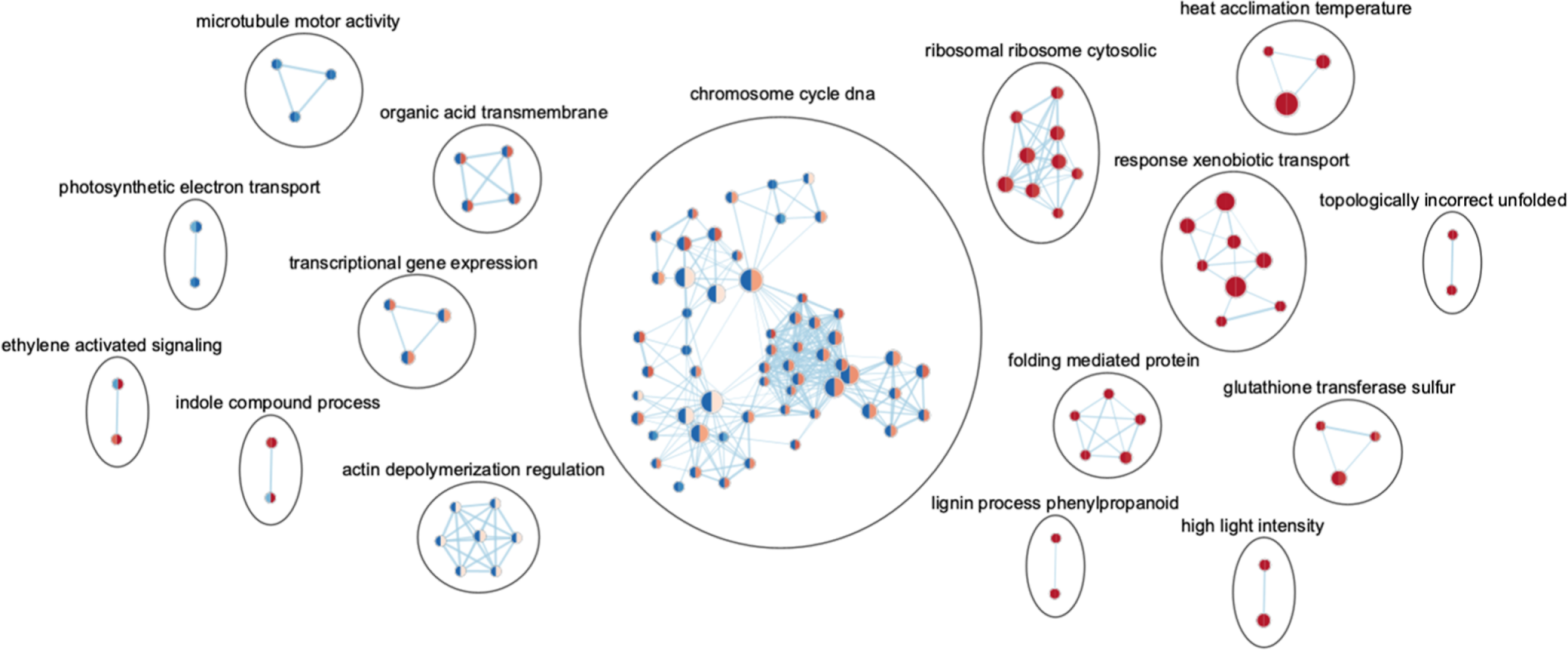
Gene Ontology (GO) enrichment analysis on gene sets from two comparison groups. Network enrichment analysis was built using all expressed genes processed using GSEA and visualized using Cytoscape v3.8.0 (FDR *q* value > 0.1). Big circles represent clusters of genes. Each cluster consists of two or more nodes (gene sets). Each node slice represents a gene set (GO term) from each comparison. Red dots represent enriched upregulated nodes, while blue dots show enriched down-regulated gene sets. Circle size is an indicator of gene number. Nodes with shared genes are connected by blue lines (edges).

**Figure 5:**
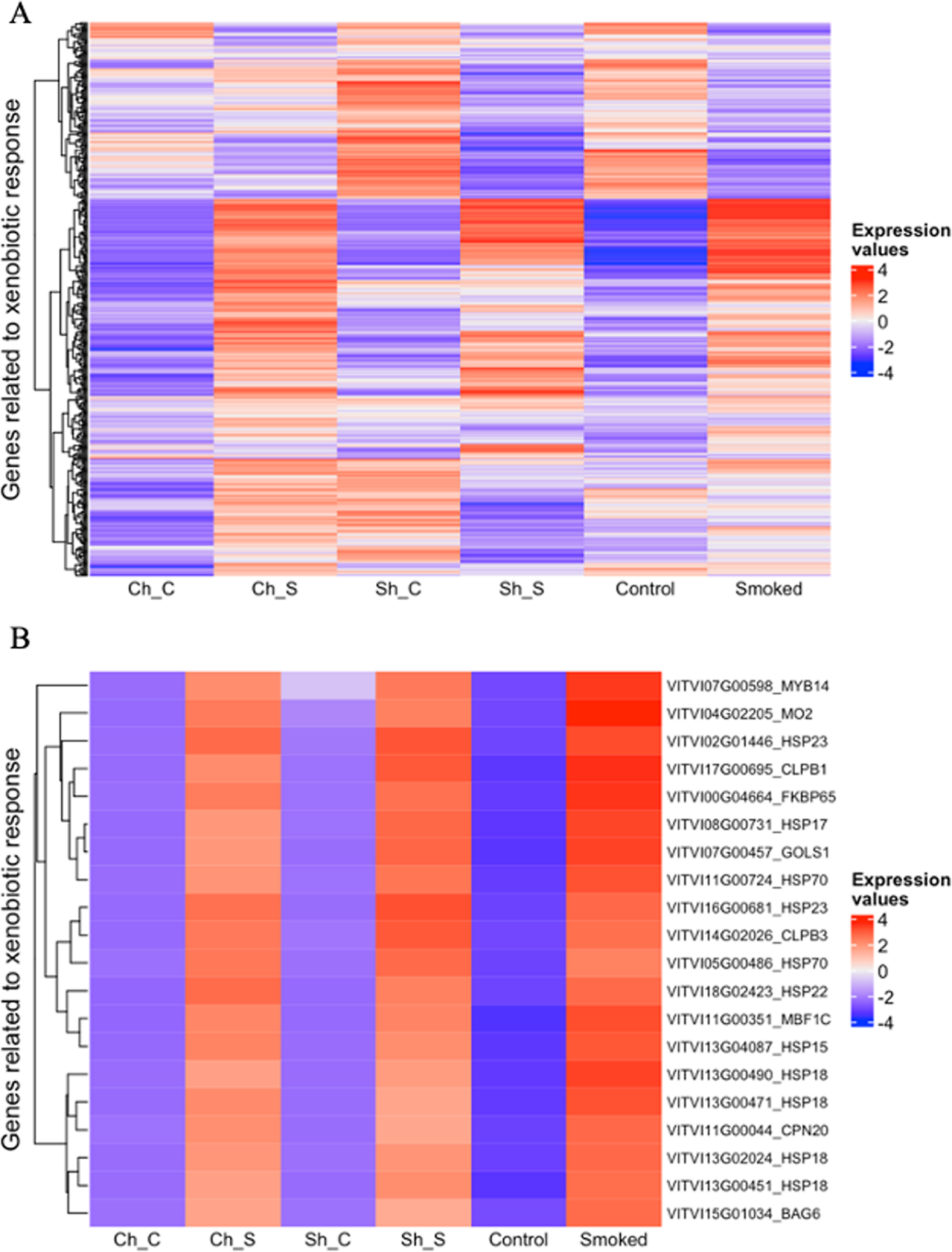
Heatmaps of genes related to xenobiotic response. (A) All xenobiotic response related genes; a total of 523 genes. (B) The 20 top-ranked candidates from GSEA using gene permutation-based test. Ch-C: Chardonnay control samples, Ch-S: Chardonnay smoked samples, Sh-C: Shiraz control samples, Sh-S: Shiraz smoked samples, control and smoked are the combined treatments from both cultivars.

### Composition of Smoke-Exposed Berries

The effect of smoke exposure on berry ripening was evaluated for each of the field-grown cultivars, i.e., Cabernet Sauvignon, Chardonnay, Merlot and Sauvignon Blanc,, by measuring total soluble solids (TSS) and berry weight (Table S7). Few statistically significant differences in TSS were observed between control and smoke exposed berries during ripening; with no significant differences in either TSS or berry weight evident at maturity. The differences that were observed were therefore attributed to natural variation rather than any impact of smoke exposure, consistent with previous research.^5^

The concentrations of phenolic glycosides used as markers of smoke taint were quantified (as syringol gentiobioside equivalents) at commercial maturity to confirm glycosylation of smoke-derived volatile phenols. Increased concentrations of several phenolic glycoconjugates were observed for smoke-exposed fruit (Table S8). Among the identified compounds, pentose glucosides of guaiacol, phenol and cresol, and the gentiobioside of syringol were the most abundant precursors, in agreement with previous studies.^7,9^

## Discussion

Grapevine smoke exposure can lead to the development of smoke taint in grapes. The uptake of smoke-derived volatile phenols, including guaiacol, cresols and syringol is followed by their glycosylation, leading to the accumulation of a pool of smoke taint precursors. To investigate the molecular consequences of smoke exposure, experiments described here sought to profile the genes that are upregulated in response to grapevine smoke exposure, and specifically, to identify glycosyltransferase genes that might encode the proteins responsible for modifying the volatile phenols that contribute to smoke taint.

RNA sequencing of berries from potted vines sampled 1 h after smoke exposure indicated significantly increased transcript levels from genes encoding HSP20 heat shock proteins (Table 1). These proteins, members of PFAM 00011, comprise a family of molecular chaperones that prevent protein aggregation under abiotic stresses.^30^ The upregulation of genes annotated in this group has been associated with the response to heat stress in grapevines.^31^ Three classes of *cis*-elements have been predicted in the promotor regions of the 48 grapevine *HSP20* genes. These are the phytohormone responsive, abiotic and biotic stress-responsive, and plant development-related *cis*-elements.^32^ The presence of these motifs supports the involvement of the Vv*HSP20* genes in a general abiotic stress response.

Here we identified 19 *VvHSP20* (PFAM00011) genes upregulated in grapes 1 h after their exposure to smoke (Table 1). Earlier work in pre- and postveraison grapevines exposed to smoke for a 36-h period and sampled at 8 time points within this period showed no up-regulation of *VvHSP20* genes.^10^ The earliest sampling in this case was 2 h after the initiation of smoke exposure. Further work should seek to determine if smoke exposure has a rapid and transient effect on the transcription of genes encoding HSP20 family members, specifically looking also at the effect of smoke exposure on the expression of Heat Stress Transcription Factor (HSF) genes. Previous work has shown that HSF family members encode transcription factors vital for the expression of heat shock genes.^33^

The expression of 5 genes encoding glycosyltransferases was upregulated following exposure to smoke in both grapevine varieties tested. Those most highly upregulated were a GT8 galactinol synthase 1, encoded by *Vitvi07g00457*, and a GT1 hydroquinone glucosyltransferase, encoded by *Vitvi15g01623* (Table 3). The involvement of galactinol synthase, also called inositol 3-α-galactosyltransferase, in abiotic stress responses is well documented. Galactinol synthases catalyze the production of raffinose family oligosaccharides (RFOs), which play a significant role in membrane protection and radical scavenging during abiotic stress conditions.^34^ Research in grapevines exposed to heat stress provided clear evidence not only for the expression of *VvGOLS1*, shown to encode a functional galactinol synthase, but also of elevated expression of *VvHsf2A*, which encodes a heat-stress transcription factor. In contrast to other HSF proteins that act as transcription factors for the heat-induced expression of genes encoding heat-shock proteins, VvHSF2A binds to promoter sequences upstream of *VvGOLS1* under heat stress conditions.^35^

Recent work in which grapes were exposed in the vineyard to smoke for up to 36 h did not report the expression of HSP20-encoding genes or sequences encoding galactinol synthase, in contrast to the data presented here.^10^ It is interesting to speculate that the earlier sampling point used in the present work, 1 h postexposure, compared to 2 h as the earliest time point used in the other work, led to the identification of transcripts that are reflective of the vine’s immediate response to the stress imposed by exposure to smoke. Experiments investigating the effect of heating bunches to 9 °C above ambient showed the transcription of *VvGOLS1* and *VvHsfA2* after 1 h of treatment, comparable with the time of sampling in the work described herein.^36^ Other factors must also be considered however, including the cultivars tested, the precise developmental timing and the intensity and duration of smoke exposure, and it remains to be determined exactly what signaling pathways are responsible for the perception and transduction of smoke-induced metabolic changes within the grape.

The GT1 UDP-glycosyltransferase family is the largest family of glycosyltransferases, and its members have been associated with glycosylation of phytohormones, phenylpropanoids, flavonoids and xenobiotics.^37^ In grapevine, 228 putative GT1 genes have been identified^38^ with several functionally characterized, including a UDP-glucose flavonoid 3-*O*-glucosyltransferase,^39^ an anthocyanin 5-*O*-glucosyltransferase^40^ and a bifunctional resveratrol/hydroxycinnamic acid glucosyltransferase.^41^

Transcripts corresponding to the putative GT1 hydroquinone glucosyltransferase gene *Vitvi15g01623* showed a 5-fold change between control and smoke treated samples for both genotypes tested (Table 3). A grapevine enzyme homologous to *hydroquinone glucosyltransferase,* UGT72B27 was identified previously^22^ and shown to have preferential activity toward volatile phenols found in smoke. Kinetic and substrate analysis indicated the highest activity in vitro against the stilbene compound resveratrol. Hydroquinone (benzene-1,4-diol or *p*-dihydroxybenzene) was not tested as a substrate, but the enzyme showed good conversion of a number of phenolic substrates isolated from smoke including 4-methylguaiacol, *m*-cresol, *o*-cresol and guaiacol. In recent work, RNA sequencing of grapevines exposed to smoke treatments at pre- and postveraison stages revealed transcription of *Vitvi15g01623* among 21 transcripts encoding GT1s in smoke-exposed berries, further supporting the data presented in our experiments.^10^

The enzyme encoded by this gene in pear (Pyrus bretschneideri) is proposed to be involved in glycosylation of hydroquinone, the final step in the biosynthesis of arbutin (4-hydroxyphenyl-β-glucopyranoside).^42^ Arbutin has strong antioxidative and membrane stabilizing properties^43^ and this specialized metabolite has been associated with responses to biotic and abiotic stresses. Drought tolerant potato cultivars have been shown to accumulate higher levels of arbutin^44^ and similar results have been reported for rice cultivars in response to combined drought and heat stresses under field conditions.^45^ It is, however, unclear if the increase in transcript levels of the hydroquinone glucosyltransferase observed in our data was a result of elevated hydroquinone levels or whether it is a defense mechanism in response to smoke exposure.

Hewitt et al. (2024)^10^ reported that genes related to detoxification, redox balance, and cell wall fortification, including *Vitvi15g01623* and *Vitvi05g04355*, were significantly upregulated following smoke exposure, the highest at 6 h postsmoke exposure in cultivar Merlot (File S8). Our experiments also detected these changes after 1 h of exposure, possibly due to the higher smoke intensity compared to Hewitt’s study. Paineau et al. (2025)^11^ further identified *Vitvi15g01623* and *Vitvi05g04355* among smoke-inducible UGTs (*VviSIUGTs*) in dwarf grapevine cultivar Pixie (File S9), consistent with our findings.

All three gene candidates identified in smoke-exposed potted vines were also expressed in field-grown samples in both control and smoke-affected fruit. Although only a single biological replicate was used for this initial analysis (due to the large sample set, comprising four cultivars, two types of tissue, and three sampling time points), distinct expression patterns emerged based on variety, tissue type and treatment (Figure 3, Table S5). Higher transcript levels were observed in berry skins, where glycosidic compounds typically accumulate.^46^ Notably, *Vitvi07g00457* consistently shows more dynamic changes, suggesting a greater response to the conditions tested, while *Vitvi05g04355* and *Vitvi15g01623* remain more stable. The differences in expression across grape varieties and tissues may reflect distinct molecular processes at play in pulp and skin during development. Supporting these observations, Table S8 shows significant increases in several phenolic glycosides in smoke-affected grapes compared to controls across multiple grape varieties. These metabolic changes align with the upregulation of genes involved in glycosylation pathways, such as UDP-glycosyltransferases (UGTs), suggesting a molecular response to smoke exposure that leads to increased phenol conjugation.

Pathway enrichment analysis identified four significantly upregulated pathways in smoke-treated Shiraz and Chardonnay. These are the heat acclimation temperature, high light intensity, response to xenobiotic transport, and lignin metabolic phenylpropanoid clusters. Except for the phenylpropanoid pathways, Galactinol synthase 1 (*Vitvi07g00457*) was the most upregulated gene in all the pathways (Files S10–S13). This observation is consistent with the suggested role of raffinose family oligosaccharides (RFOs), synthesized by galactinol synthases, as signaling molecules which play a significant role in membrane protection and radical scavenging during abiotic stress conditions.^34^ Overexpression of two galactinol synthases, *AtGolS2* and *AtGolS3,* enhanced salt tolerance in transgenic poplar and resulted in higher levels of soluble sugars and other stress-response related metabolites such as proline and salicylic acid.^47^

The heat acclimation temperature and high light intensity pathways shared ten upregulated *HSP20* genes (Files S11 and S12). As stated, the *HSP20* genes are stress-response heat shock proteins. The change in temperature between untreated and treated grapes in our experiment was less than 2 °C with an ambient temperature of 27 °C. Thus, the upregulation of these two pathways may not necessarily be related to an increase in temperature or light intensity during smoke exposure but rather to a common stress response. Additionally, multiple genes from the phenylpropanoid pathway were upregulated in response to smoke exposure. This may be due to the antioxidant properties and the protective effect of phenolics and flavonoids against the free radical oxygen species formed during the exposure of plants to stress conditions. Also, evidence exists that the uptake of volatile phenols by grapevines after exposure to smoke could alter the endogenous metabolism of these compounds.^48^ Upregulation of the xenobiotic response pathway in response to smoke exposure is not surprising since xenobiotic transporters could potentially be involved in transport or compartmentation of xenobiotic conjugates. However, transporters capable of moving glycosylated volatile phenols have not been identified to date. Potentially, there is a dual role of the xenobiotic transporters that could be explored further.

In contrast to many of the most highly upregulated genes belonging to one PFAM, downregulated genes showed variability in their predicted functions. The most downregulated gene for both Chardonnay and Shiraz is predicted to come from the expansin family. These proteins are implicated in cell wall expansion and are highly regulated during berry development, with different expansins being expressed at various developmental stages.^49^ These proteins are thought to be downregulated in response to abiotic stresses, including heat and drought, to halt cell division and growth.^50^ In grapevine cultivars such as Touriga Nacional and Trincadeira, the expression of β-expansin was found to be downregulated in response to heat and light radiation stresses.^51^ It is possible that the downregulation of expansin gene expression in smoke-affected berries triggers an analogous response, linked to a temporary halt in berry expansion, and this could be measured in future experiments.

With the increasing intensity and frequency of bushfires and their damaging effects on fruit quality, it is more important than ever to understand the response of plants to smoke exposure. Our RNA-Seq data revealed the upregulation of common stress response pathways in smoke-exposed berries of both Chardonnay and Shiraz. Genes encoding the small heat shock proteins HSP20s were the most upregulated, followed by two glycosyltransferases from the GT8 and GT1 families. While most previous studies have focused on GT1 genes as the primary candidates induced by smoke exposure, our findings suggest that other glycosyltransferase families, such as GT8, may also play important roles and warrant further investigation. A more comprehensive understanding of the full spectrum of GT involvement will be critical for developing effective strategies to manage and mitigate smoke taint. There is also scope to further explore the identity of transporters that may be translocating glycosylated volatile phenols, with some suggestion that these may be one and the same as those moving xenobiotic compounds, while effects of smoke on berry expansion may also prove informative.

## Supplementary Material





## Notes

Table S1. RNA sequencing results as supplied by AGRF. 100bp Single End -Flowcell ID: C9P2AANXX. Table S2. List of tools and databases. Table S3. Summary of RNA-seq read processing and mapping statistics for Shiraz and Chardonnay samples under control and smoke-affected conditions. Table S4. Primer sequences used for qRT-PCR analysis of selected grapevine genes. Table S5. Expression levels of candidate genes in control (c) and smoke-exposed (s) grape berries. Table S6. Clusters of enriched Gene Ontology (GO) terms in smoke-exposed Shiraz and Chardonnay grapes. Table S7. Total soluble solids (TSS) and berry weight of grapes sampled from control (C) and smoke-affected (S) grapevines at different time points. Table S8. Concentrations of phenolic glycosides (μg/kg) in grapes from control (C) and smoke-exposed (S) grapevines (PDF)
File S1. Predicted function for V. vinifera genes. File S2. Functional annotation of V. vinifera genes using EggNOG mapper. File S3. Database of V. vinifera GO terms. File S4. Distribution of Glycosyltransferase (GT) family genes identified in V. vinifera based on EggNOG-mapper results. File S5. Orthogroup assignments of all protein coding genes in V. vinifera, A. thaliana, and Pyrus × bretschneideri using OrthoFinder. File S6. RNA sequencing results of smoked vs control samples of Shiraz and Chardonnay grapes presented as Counts Per Million (CPM). File S7. Log fold change (Log FC) of differentially expressed gene (DGE) of six pairwise comparisons Shiraz and Chardonnay grapes. File S8. Differential expression (log FC) of genes identified by Hewitt et al. (2024)^10^ (V. vinifera cv. Merlot) in Shiraz (Sh) and Chardonnay (Ch) grapes following smoke exposure. Log FC values represent expression changes in smoke-treated berries compared to controls. Yellow highlights indicate genes that were also included as candidate genes in our study (Table 1 and Table 3). File S9. Identification of homologous glycosyltransferase genes between two reference genomes of V. vinifera. Red highlights indicate genes that are among the 12 GT1 genes identified in the study by Paineau et al. (2025)^11^ File S10. Genes in the xenobiotic response pathway identified through the pathway enrichment analysis. File S11. Genes in the heat acclimation temperature pathway identified through the pathway enrichment analysis File S12. Genes in high-light intensity pathway identified through the pathway enrichment analysis. File S13. Genes in the lignin metabolic phenylpropanoid pathway identified through the pathway enrichment analysis (XLSX)

## Abbreviations

CAZy: carbohydrate-active enZymes database
CPM: counts per million
GT: glycosyltransferase
GO: gene ontology
cv: cultivar
GolS: galactinol synthase
HqGT1: Hydroquinone glucosyltransferase
HPLC-MS/MS: high performance liquid chromatography-tandem mass spectrometry
