# Supplementary material for: Transcription Profiling of Glycosyltransferases in Vitis vinifera Cultivars Following Smoke Exposure
Source: J Agric Food Chem. 2025 Aug 19;73(35):22046–58. doi: 10.1021/acs.jafc.5c06076 (PMC12412534; doi:10.1021/acs.jafc.5c06076)
Supplement: Supplementary file 1 [file jf5c06076_si_001.pdf]

Supporting Information

Transcription Profiling of Glycosyltransferases in *Vitis vinifera* Cultivars Following Smoke Exposure

Lieke van der Hulst[1,2], Lina Herliana[3], Ali Saleh Hassan[1], Julian G. Schwerdt[1,4], Neil Shirley[1,4], Christopher M. Ford[1*], Rachel A. Burton[1,4*], Kerry L. Wilkinson[1,2]

[1] School of Agriculture, Food and Wine, Waite Research Institute, The University of Adelaide, PMB 1, Glen Osmond, South Australia, 5064

[2] The Australian Research Council Training Centre for Innovative Wine Production, Glen Osmond, South Australia, 5064

[3] Research Center for Genetic Engineering, National Research and Innovation Agency (BRIN), Bogor, West Java, 16911, Indonesia

[4] The Australian Research Council Centre of Excellence in Plant Cell Walls, PMB 1, Glen Osmond, South Australia, 5064

* Corresponding Authors: Associate Professor Christopher Ford, telephone: + 61 8 8313 7386, facsimile: + 61 8 8313 7716, email: christopher.ford@adelaide.edu.au; and Professor Rachel Burton, telephone: + 61 8 8313 1057, facsimile: + 61 8 8313 7716, email: rachel.burton@adelaide.edu.au

Table S1. RNA sequencing results as Supplied by AAGRF . 100bp Single End - Flowcell ID: C9P2AANXX.

Lane	Sample Name	Single Reads	Data Yield (bp)
3	1_Sh_C1	18,108,940	1.81 Gb
	2_Sh_C2	19,071,909	1.91 Gb
	3_Sh_C3	18,275,556	1.83 Gb
	4_Sh_S1	20,413,483	2.04 Gb
	5_Sh_S2	16,620,576	1.66 Gb
	6_Sh_S3	18,166,211	1.82 Gb
	7_Ch_C1	14,864,484	1.49 Gb
	8_Ch_C2	14,260,737	1.43 Gb
	9_Ch_C3	18,846,957	1.88 Gb
	10_Ch_C1	17,912,594	1.79 Gb
	11_Ch_C2	21,945,092	2.19 Gb
	12_Ch_C3	19,172,783	1.92 Gb
	Total	217,659,322	21.77 Gb

Sh = Shiraz; Ch = Chardonnay; C = Control; S = Smoke-affected samples.

Table S2. List of tools and databases.

Name	References
BLAST v2.12.0	Madden, T., 2013. The BLAST sequence analysis tool. *The NCBI handbook*, *2*(5), pp.425-436.
MAKER v2.31.11	Cantarel, B.L., Korf, I., Robb, S.M., Parra, G., Ross, E., Moore, B., Holt, C., Alvarado, A.S. and Yandell, M., 2008. MAKER: an easy-to-use annotation pipeline designed for emerging model organism genomes. *Genome research*, *18*(1), pp.188-196.
eggNOG-mapper	Cantalapiedra, C.P., Hernández-Plaza, A., Letunic, I., Bork, P. and Huerta-Cepas, J., 2021. eggNOG-mapper v2: functional annotation, orthology assignments, and domain prediction at the metagenomic scale. *Molecular biology and evolution*, *38*(12), pp.5825-5829. http://eggnog-mapper.embl.de
OrthoFinder v2.5.5	Emms, D.M. and Kelly, S., 2019. OrthoFinder: phylogenetic orthology inference for comparative genomics. *Genome biology*, *20*, pp.1-14.
FastQC v0.11.9	https://www.bioinformatics.babraham.ac.uk/projects/fastqc/
MultiQC v1.9	Ewels, P., Magnusson, M., Lundin, S. and Käller, M., 2016. MultiQC: summarize analysis results for multiple tools and samples in a single report. *Bioinformatics*, *32*(19), pp.3047-3048.
Trimmomatic v0.39	Bolger, A.M., Lohse, M. and Usadel, B., 2014. Trimmomatic: a flexible trimmer for Illumina sequence data. *Bioinformatics*, *30*(15), pp.2114-2120.
BBDuk, Bbmap v.38.87	Bushnell, B., 2014. BBMap: a fast, accurate, splice-aware aligner.
STAR v2.7.10b	Dobin, A. and Gingeras, T.R., 2015. Mapping RNA-seq reads with STAR. *Current protocols in bioinformatics*, *51*(1), pp.11-14.
featureCounts (subreads v2.01)	Liao, Y., Smyth, G.K. and Shi, W., 2014. featureCounts: an efficient general purpose program for assigning sequence reads to genomic features. *Bioinformatics*, *30*(7), pp.923-930.
edgeR	Robinson, M.D., McCarthy, D.J. and Smyth, G.K., 2010. edgeR: a Bioconductor package for differential expression analysis of digital gene expression data. *bioinformatics*, *26*(1), pp.139-140.
factoextra R package	Kassambara, A. and Mundt, F., 2017. Package 'factoextra'. *Extract and visualize the results of multivariate data analyses*, *76*(2), pp.10-18637.
limma package	Ritchie, M.E., Phipson, B., Wu, D.I., Hu, Y., Law, C.W., Shi, W. and Smyth, G.K., 2015. limma powers differential expression analyses for RNA-sequencing and microarray studies. *Nucleic acids research*, *43*(7), pp.e47-e47.
VennDiagram R package	Chen, H. and Boutros, P.C., 2011. VennDiagram: a package for the generation of highly-customizable Venn and Euler diagrams in R. *BMC bioinformatics*, *12*, pp.1-7.

GSEA (Gene Set Enrichment Analysis).	Shi, J. and Walker, M.G., 2007. Gene set enrichment analysis (GSEA) for interpreting gene expression profiles. *Current Bioinformatics*, *2*(2), pp.133-137.
V. vinifera PN40024.V4	https://plants.ensembl.org/Vitis_vinifera/Info/Annotation/#assembly
UniProtKb database	https://www.uniprot.org/taxonomy/33090
TAIR10 20101214	https://www.arabidopsis.org
Pyrus × bretschneideri genome v1.1	https://www.rosaceae.org
SILVA database	https://www.arb-silva.de/fileadmin/silva_databases/release_128/Exports/SILVA_128_LSURef_tax_silva.fasta.gz<br>https://www.arb-silva.de/fileadmin/silva_databases/release_138/Exports/SILVA_138_SSURef_NR99_tax_silva.fasta.gz<br>https://www.arb-silva.de/fileadmin/silva_databases/release_138/Exports/SILVA_138_SSURef_tax_silva.fasta.gz
5S rRNA	http://combio.pl/rrna/static/download/Archaea.fasta<br>http://combio.pl/rrna/static/download/Bacteria.fasta<br>http://combio.pl/rrna/static/download/Mitochondria.fasta<br>http://combio.pl/rrna/static/download/Plastids.fasta<br>http://combio.pl/rrna/static/download/Eukaryota.fasta
Vitis vinifera cv. Pixie cl. FPS01	https://grapegenomics.com/pages/VvPixie/blast.php

Table S3. Summary of RNA-Seq read processing and mapping statistics for Shiraz and Chardonnay samples under control and smoke-affected conditions.

Samples	Raw	Trimmed	Clean	Uniquely mapped		Multiple mapped	
	M Seqs				%	Seqs	%
Sh_C1	18.1	18.0	17.4	16.5	95.10	821,511	0.20
Sh_C2	19.1	19.0	18.6	17.7	95.30	850,602	0.10
Sh_C3	18.3	18.2	17.9	17.0	95.10	848,139	0.20
Sh_S1	20.4	20.3	19.8	18.8	95.00	959,178	0.10
Sh_S2	16.6	16.6	16.1	15.3	95.00	777,137	0.10
Sh_S3	18.2	18.1	17.6	16.7	94.70	902,887	0.20
Ch_C1	14.9	14.8	14.4	13.7	95.20	666,538	0.20
Ch_C2	14.3	14.2	13.8	13.2	96.00	524,321	0.20
Ch_C3	18.8	18.8	18.2	17.4	95.50	789,624	0.10
Ch_C1	17.9	17.8	17.3	16.4	95.20	793,753	0.20
Ch_C2	21.9	21.8	21.2	20.2	95.40	926,965	0.20
Ch_C3	19.2	19.1	18.4	17.6	95.50	814,232	0.10

Sh = Shiraz; Ch = Chardonnay; C = Control samples; S = Smoke-affected samples;
1-3 = Biological replicate number; M Seqs = Million Sequences.

Table S4. Primer sequences used for qRT-PCR analysis of selected grapevine genes.

Gene IDs	Forward Primer	Reverse Primer	Size (bp)	Acquisition temperature
Vitvi07g04656	CAGCCAGATCTCAGCAGGCTT	GTTCTCGGCAGATTGACCATTA	119	79
Vitvi07g04157	CCATGGCATCATCACCACAA	GTCAACATCAGTCACTGGTACAAAACC	72	80
Vitvi04g01613	GCATCCCTCAGCACCTTCC	AACCACCCACTCACAATCCTCC	203	80
Vitvi16g01364	AGTAGATGACTGGGATTGGAGGT	GAGTATCAACAAAAAGAAAGCTG	177	80
Vitvi07g00457	TGAATTTGGAGCCTTTTCATTG	ACCCCCACAAAACAAAAAAATAA	187	79
Vitvi15g01623	GAAAGGAGTGATACGGCATCGAA	TGTGAGCCTTCCCATTTTGAG	117	79
Vitvi05g04355	AAGAGGTGGTCTAGACTTGGTCC	TCAGATGTTGACTCACCTCCACCT	115	78

Vitvi07g04656 (Tubulin), *Vitvi07g04157* (Malate dehydrogenase), *Vitvi04g01613* (Actin), *Vitvi16g01364* (Ubiquitin), *Vitvi07g00457 (GOLS1 Galactinol synthase 1, GT8), Vitvi15g01623 (Hydroquinone glucosyltransferase, GT1), and Vitvi05g04355* (putative UGT75L17 Phloretin 4'-*O*-glucosyltransferase, GT1).

Table S5. Expression levels of candidate genes in control (c) and smoke-exposed (s) grape berries.

Cultivar	Tissue	Treatment	Sample time	*Vitvi07900457*	*Vitvi15901623*	*Vitvi05904355*
Chardonnay	skin	c	t = 0	325	1912	173
			t = 1	240	2256	79
			t = 7	645	1423	161
		s	t = 0	429	3917	161
			t = 1	347	3676	70
			t = 7	407	1317	95
	pulp	c	t = 0	687	2926	104
			t = 1	180	1707	30
			t = 7	194	991	24
		s	t = 0	491	2280	159
			t = 1	256	3892	104
			t = 7	240	469	49
Sauvignon Blanc	skin	c	t = 0	177	4608	171
			t = 1	148	1235	95
			t = 7	4984	1401	132
		s	t = 0	133	1761	67
			t = 1	99	1700	74
			t = 7	3120	512	108
	pulp	c	t = 0	191	2095	38
			t = 1	100	777	63
			t = 7	1231	763	47
		s	t = 0	96	2009	21
			t = 1	45	753	14
			t = 7	829	212	51
Cabernet Sauvignon	skin	c	t = 0	254	398	107
			t = 1	892	479	57
			t = 7	20353	489	80
		s	t = 0	208	2745	727
			t = 1	638	463	55
			t = 7	8972	483	66
	pulp	c	t = 0	108	311	23
			t = 1	436	271	14
			t = 7	11781	272	40
		s	t = 0	58	378	17
			t = 1	405	437	31
			t = 7	6650	395	69

Merlot	skin	c	t = 0	182	836	84
			t = 1	9993	459	86
			t = 7	354	270	100
		s	t = 0	421	2095	1265
			t = 1	12204	594	137
			t = 7	423	304	49
	pulp	c	t = 0	86	515	17
			t = 1	8174	329	24
			t = 7	257	120	13
		s	t = 0	125	739	14
			t = 1	9396	824	30
			t = 7	581	255	10

Table S6. Clusters of enriched Gene Ontology (GO) terms in smoke-exposed Shiraz and Chardonnay grapes.

	Shiraz	Chardonnay
Chromosome cycle DNA	58	-
Ribosomal ribosome cytosolic	9	-
Response xenobiotic transport	8	3
Actin depolymerization regulation	7	-
Amino sulfur glutathione	3	-
Folding chaperone mediated	5	-
Organic transmembrane activity	4	-
Heat acclimation temperature	3	2
Microtubule motor activity	3	-
Transcriptional gene expression	3	-
Ethylene activated signalling	-	2
High light intensity	2	1
Indole compound metabolic	-	2
Lignin metabolic phenylpropanoid	2	1
Photosynthetic electron transport	-	2
Topologically incorrect unfolded	2	-

Numbers represent the count of genes associated with each GO term in the respective cultivar. GO terms highlighted in yellow are shared between Shiraz and Chardonnay.

Table S7. Total soluble solids (TSS) and berry weight of grapes sampled from control (C) and smoke-exposed (S) grapevines at different time points.

		TSS (° Brix)				Berry weight (g)
		t = 0	t = 1	t =7	maturity[†]	maturity[†]
Chardonnay	C	13.0	14.6	15.6	23.2	1.4
	S	13.5	15.7	17.1	22.7	1.5
	P	*ns*	*ns*	*0.031*	*ns*	*ns*
Sauvignon Blanc	C	13.5	10.9	16.7	13.5	1.2
	S	13	12.9	17.9	13	1.3
	P	*ns*	*ns*	*ns*	*ns*	*ns*
Cabernet Sauvignon	C	12.9	13.4	14.5	12.9	1.1
	S	13.6	12.7	14.7	13.6	1.1
	P	*ns*	*ns*	*ns*	*ns*	*ns*
Merlot	C	16.4	15.8	17.5 b	16.4	1.7
	S	15.3	15.3	16.4 a	15.3	1.7
	P	*ns*	*ns*	*0.037*	*ns*	*ns*

Sample times are reported as days after smoke exposure. [†] Maturity corresponds to t = 17, t = 28, t = 28 and t = 22 days for Chardonnay, Sauvignon Blanc, Cabernet Sauvignon and Merlot, respectively. Values represent means of three replicates (n = 3). P values indicate significant differences between treatments ($P < 0.05$); ns = not significant.

Table S8 Concentrations of phenolic glycosides (μg/kg) in grapes from control (C) and smoke-exposed (S) grapevines.

Treatment		GuR	GuGB	GuPG	GuG	CrR	CrPG	PhR	PhGB	PhPG	SyrGB	4MGR	4MGPG	4MSGB
Chardonnay	C	tr	tr	2 b	1	tr	4 b	tr	tr	6 b	1 b	tr	3	tr
	S	tr	tr	12 a	2	2	9 a	1a	1a	13 a	24 a	2	3	2
	P	*ns*	*ns*	*0.016*	*ns*	-	*0.038*	-	-	*0.005*	*0.033*	-	*ns*	-
Sauvignon Blanc	C	tr	tr	1	1	tr	2	tr	tr	3	1	tr	tr	tr
	S	10	23	13	4	25	11	11	1	24	33	10	tr	6
	P	-	-	*0.003*	*0*	-	*0.004*	-	-	*<0.001*	*<0.001*	-	*ns*	-
Cabernet Sauvignon	C	tr	tr	2	1	1	2	tr	tr	4	2	tr	2	tr
	S	7	4	27	3	14	13	7	3	26	59	5	2	5
	P	-	-	*ns*	*ns*	*ns*	*ns*	-	-	*ns*	*ns*	-	*ns*	-
Merlot	C	tr	tr	3	1	tr	4	tr	tr	7	2	tr	3	tr
	S	3	24	77	10	9	43	4	3	56	66	5	4	9
	P	-	-	*ns*	*ns*	*ns*	*ns*	-	-	*ns*	*ns*	-	*ns*	-

Values represent the mean of three replicates; tr = trace (i.e., 0.5-1 μg/L). Different letters within columns (for each variety) indicate statistical significance (P = 0.05, one-way ANOVA); ns = not significant; Gu = guaiacol; Cr = cresol; Ph = phenol; Syr = syringol; 4MG = 4-methylguaiacol; 4MS = 4-methylsyringol; R = rutinoside; GB = gentiobioside; PG = pentose glucoside; G = glucoside.
